# Supplementary material for: The morphology and internal structure of dogwood (Cornus L.) endocarps in the taxonomy and phylogeny of the genus
Source: PeerJ. 2021 Oct 28;9:e12170. doi: 10.7717/peerj.12170 (PMC8557701; doi:10.7717/peerj.12170)
Supplement: Supplemental Information 1 [file peerj-09-12170-s001.docx]

List of the botanical collections from which the cultivated materials were collected

| No | Names of arboreta/botanical gardens | Locality | Latitudes  longitudes |
| --- | --- | --- | --- |
| 1 | Adam Mickiewicz University Botanical Garden in Poznań | Poznań, PL | 52° 25’ N,  16° 53’ E |
| 2 | Bolestraszyce Arboretum | Bolestraszyce, PL | 49° 49' N,  22° 51' E |
| 3 | Botanic Garden of the Jagiellonian University | Kraków, PL | 50° 04’ N,  19° 58’ E |
| 4 | Botanical Garden of University of Coimbra | Coimbra, PT | 40° 12’ N,  08° 25’ E |
| 5 | Dendrological Garden in Glinna | Glinna, PL | 53° 17’ N,  14° 43’ E |
| 6 | Dendrological Garden of University of Life Sciences in Poznań | Poznań, PL | 52° 25’ N,  16° 53’ E |
| 7 | Gołubie Botanical Garden | Gołubie, PL | 54° 21’ N,  18° 02’ E |
| 8 | Higher Institute of Agronomy ISA, University of Lisbon | Lisbon, PT | 38° 42’ N,  09° 10’ E |
| 9 | Kalmthout Arboretum | Kalmthout, BE | 51° 00’ N,  04° 25’ E |
| 10 | Kórnik Arboretum | Kórnik, PL | 52° 14' N,  17° 05' E |
| 11 | Longwood Gardens | Kennett Square, US | 39° 52’ N,  75° 40’ E |
| 12 | National Botanic Gardens | Dublin, IE | 55° 02’ N,  07° 38’ E |
| 13 | New York Botanical Garden | New York, US | 40° 51’ N,  73° 52’ E |
| 14 | Research Center for Medicinal Plant Resources, Nat. Institute of Biomedical Innovation | Hokkaido, JP | 44° 22' N,  142° 26' E |
| 15 | The Botanic Garden of Smith College | Northhampton, US | 49° 12’ N,  72° 38’ E |
| 16 | Warsaw University of Life Sciences (SGGW) Rogów Arboretum | Rogów, PL | 51° 49' N,  19° 53' E |
| 17 | Wojsławice Arboretum | Wojsławice, PL | 50° 43’ N,  16° 51’ E |
| 18 | Wroclaw University Botanical Garden | Wrocław, PL | 51° 11’ N,  17° 04’ E |

Abbreviations: BE – Belgium, IE – Ireland, JP – Japan, PL – Poland, PT – Portugal, US – United States
