# Supplementary material for: The morphology and internal structure of dogwood (Cornus L.) endocarps in the taxonomy and phylogeny of the genus
Source: PeerJ. 2021 Oct 28;9:e12170. doi: 10.7717/peerj.12170 (PMC8557701; doi:10.7717/peerj.12170)
Supplement: Supplemental Information 2 [file peerj-09-12170-s002.docx]

The origin of the cultivated materials and their taxonomic verification

| No. of specimen | No. of collection | No. in collection/ no. in IS.^1^ (coordinates or name of parcel in collection) | Origin of specimens:  type of locality^2^,  type of reproduction^3^, obtained as/from^4^, date since it was cultivated | Species identification verified by | Verification based on |
| --- | --- | --- | --- | --- | --- |
| *C. mas* | | | | | |
| 1 | 5* | 530 (9) | C^2^, 2000/2002 | AW | FR^6^, LV^6^ |
| 2 | 10 | 49 (6) | No data | AW | FR, LV |
| 3 | 16 | 10792 (4/2) | C, GR^3^, NL^2^, 1982/1993 | AW | FR, LV |
| 4 | 6 | (12) | No data | AW | FR, LV |
| 5 | 1 | 1519 (D18) | No data | AW | FR, LV |
| 6 | 4 | IS. 2010 PTOCOI 508 | No data | OD^5^ | No data |
| *C. officinalis* | | | | | |
| 1 | 3 | 49/9 | C, GR, P^4^, 1979 | AW | FR, LV |
| 2 | 10 | 6956 (3) | C, 1935 | AW | FR, LV |
| 3 | 10 | 6956 (3) | C, 1935 | AW | FR, LV |
| 4 | 1 | 4987 (D26) | C, GR, S^4^, 1995/2000 | AW | FR, LV |
| 5 | 11 | No data | No data | OD | No data |
| 6 | 9 | IS. 2010 4015 | No data | OD | No data |
| *C. florida* | | | | | |
| 1 | 18 | 3709 | C | AW | FR, LV |
| 2 | 2 | 1698 | No data | AW | FR, LV |
| 3 | 13 | NYBG78784*DF | No data | OD | No data |
| 4 | 13 | NYBG78784*EB | No data | OD | No data |
| 5 | 9 | IS. 2010 91112 A | No data | OD | No data |
| 6 | 8 | No data | No data | OD | No data |
| *C. kousa* | | | | | |
| 1 | 1 | 740 (G10) | C, P, 1949 | AW | FR, LV |
| 2 | 17 | 3710 | S | AW | FR, LV |
| 3 | 3 | (41/19) | C, P, 1985 | OD | No data |
| 4 | 6 | (18) | No data | AW | FR, LV |
| 5 | 5 | 125 | NL, 1971/1972 | AW | FR, LV |
| 6 | 7 | No data | No data | OD | No data |
| *C. nuttallii* | | | | | |
| 1 | 12 | No data | No data | OD | No data |
| *C. canadensis* | | | | | |
| 1 | 15 | 39 | NL, GR | OD | No data |
| 2 | 18 | 2453 | C | AW | FR, LV |
| 3 | 14 | IS. 2011 90 | No data | OD | No data |

*- numbers according to Table 2

Abbreviations: ^1^ IS. – Index Seminum, ^2^ type of locality: C – cultivated plant, NL - natural locality; ^3^ type of reproduction: GR - generative reproduction, VR – vegetative reproduction; ^4^ obtained as: CT - cuttings, P - plant, S - seeds; ^5^ verification of species determination: AW – A. Woźnicka, OD – original determination; ^6^ distinguishing features: FL – flowers, FR – fruits, IF – inflorescences, LV – leaves;
