# Supplementary material for: The morphology and internal structure of dogwood (Cornus L.) endocarps in the taxonomy and phylogeny of the genus
Source: PeerJ. 2021 Oct 28;9:e12170. doi: 10.7717/peerj.12170 (PMC8557701; doi:10.7717/peerj.12170)
Supplement: Supplemental Information 3 [file peerj-09-12170-s003.docx]

The provenance and the geographic origin of the *Cornus* species herbarium accessions tested

| No of specimen | *Cornus* species | *Cornus s*pecies according to herbarium sheet | International symbol of herbarium / name of plant collection | No of  herbarium sheet | Locality | Latitudes,  longitudes | Collection date | Collector | Author of determination/verification |
| --- | --- | --- | --- | --- | --- | --- | --- | --- | --- |
| 1 | *mas* | *mas* L. | KOR * | 12955 | SK, river Hron | 48°35' N, 18°52' E | 18.09.1959 | K. Browicz | -/AW |
| 2 |  | *mas* L. | KOR | 12960 | SI, Kamnickie Alps, river Kamniška Bistrica | 46°17' N, 14°35' E | 29.09.1960 | - | -/AW |
| 3 |  | *mas* L. | H | 1016149 | BA, Mostar, Cim | 43°21' N, 17°46' E | 30.05.1905 | H. Lindberg | H. Lindberg/  AW |
| 4 |  | *mas* L. | H | 1748099 | CS, massif Rtanj, Tumba mountain | 43°42' N, 21°57' E | 14.09.2009 | P. Uotila | -/AW |
| 5 |  | *mas* L. | G | G00308521 (24708) | GR, Kristallopigi | 40°38' N, 21°05' E | 18.07.1985 | A. Strid | -/AW |
| 6 | *officinalis* | *officinalis* S.J. | BM | BM001124455 (6246) | JP, Hirosaki | 40°35' N, 140°27' E | 1904 | B. Faurie | -/AW |
| 7 |  | *officinalis* | K | 79 | KR | 38°18' N, 127°09' E | 28.09.1982 | Beyer, Erskine, Cowley | - |
| 8 |  | *officinalis* | BM | BM001124456 (8750) | KR, Keijo | 37°36' N, 126°58' E | 06.07.1917 | E. H. Wilson | -/AW |
| 9 |  | *officinalis* | K | 15.07.2002 | CN | 39°50' N, 135°00' E | 15.07.2002 | Xu Ren-xin | - |
| 10 | *florida* | *florida* L. | H | 1140451 (31) | US, Teksas, Athens | 32°12' N, 95°48' W | 24.11.1973 | C. Mathis | -/AW |
| 11 |  | *florida* L. | L | 932369 | US, New Jersey, Princeton, Herrontown Woods | 40°22' N, 74°38' W | 07.10.1991 | F. Hekker | -/AW |
| 12 |  | *florida* L. | KRAM | 2001632 | US, Michigan, Baker Woodlot | 42°42' N, 84°28' W | 05.10.1069 | W. D. Stevens | - |
| 13 |  | *florida* L. | KRAM | 93986 | US, Maryland, Loch Raven Reservoir | 39°27' N, 76°34' W | 24.08.1970 | Windler, Keenan, Lombardo, Williams | - |
| 14 | *kousa* | *kousa* Buerg. ex Hance | L | 789596 | JP, prefecture Fukui, Nanjo-gun | 35°46' N, 136°12' E | 28.08.1973 | H. Kanai | -/AW |
| 15 |  | *kousa* Hance. | KOR | 28343 | CN, Shaanxi province, Taibai Mountain | 33°58' N, 107°48' W | 09.09.1959 | S. Białobok | -/AW |
| 16 |  | *kousa* Buerg. | KOR | 12953 | CN, Henan province, Lao-chün Shan Mountain | 33°43' N, 111°38' E | 06.09.1935 | K. M. Liou | T. P. Wang/  AW |
| 17 |  | *kousa* F. Buerger ex Hance | S | S13-11977 | JP, prefecture Nagano, Matsubara lake | 36°03' N, 138°27' E | 25.09.1958 | M. Furuse | - |
| 18 | *nuttalii* | *nuttalii* Audubon ex Torr. & A. Gray | S | S13-11985 | US, South California, San Bernardino | 34°07' N, 117°17' W | 03.09.1931 | B. C. Templeton | - |
| 19 |  | *nuttalii* Audubon ex Torr. & A. Gray | S | S13-11986 | US, California, Humboldt | 40°49' N, 123°52' W | 15.08.1939 | Law | - |
| 20 |  | *nuttalii* | H | 1058531 | CA, British Columbia, Victoria, Goldstream | 48°27' N, 123°33' W | 11.-12.09.1931 | V. Kujala,  A. Cajander | -/AW |
| 21 |  | *nuttalii* Aud. | L | 789587 | US, California, Mariposa Grove | 37°30' N, 119°35' W | 07.08.1939 | C. Skottsberg, H. J. Lam, H. P. Bracelin | -/AW |
| 22 |  | *nuttalii* Aud. | L | 789586 | US, California, Robert Louis Stevenson State Park | 38°39' N, 122°36' W | 10.09.1967 | F. R. Fosberg | -/AW |
| 23 | *canadensis* | *canadensis* L. | KOR | 23987 | RU, Amur district, Byssy river-basin | 52°23' N, 131°57' E | 17.08.1927 | O. Kuzenewa | -/AW |
| 24 |  | *canadensis* | KOR | 47445 | US, Minnesota, Straight Lake | 46°56' N, 95°16' W | 24.07.1981 | - | -/AW |
| 25 |  | *canadensis* L. | H | 1399488 (6739) | CA, Ontario, Thunder Bay district, St. Ignace Island | 48°47' N, 87°55' W | 07.08.1959 | C. E. Garton | -/AW |
| 26 |  | *canadensis* L. | H | 1399506 (192) | CA, Quebec, Montcalm | 46°47' N, 71°13' W | 03.08.1953 | J. Rousseau | -/AW |
| 27 |  | *canadensis* L. | L | 932398 | CA, Ontario, Deep River | 46°06' N, 77°29' W | 16.-21.06.1965 | M. I. Moore | -/AW |
| 28 |  | *canadensis* L. | L | 932399 | CA, Ontario, St. Joseph Island | 46°18' N, 83°58' W | 15.08.2002 | D. Goldman | -/AW |
| 29 |  | *canadensis* L. | H | 1016146 | CA, Ontario, Thunder Bay district, Black Sturgeon Lake | 49°21' N, 88°51' W | 1.-12.08.1956 | H. Lindberg | -/AW |
| 30 |  | *canadensis* | L | 789607 | US, Maryland, Ellicott City | 39°16' N, 76°47' W | 09.1958 | Gloor | Gloor/AW |
| 31 |  | *canadensis* L. | KRAM | 13263 | CA, Saskatchewan, Flotten Lake | 54°36' N, 108°31' W | 13.08.1969 | J. Looman | -/AW |
| 32 | *suecica* | - | BG1 | - | SE, Jämtland, Enaforsholm | 63°16' N, 12°19' E | 2010 | No data | - |
| 33 |  | *suecica* L. | GH | 46,682 | CA, Quebec, Kamouraska | 47°33' N, 69°51' W | 06.08.1936 | F. Marie-Victorin, F. Rolland-Germain,  F. Dominique | -/AW |
| 34 |  | *suecica* L. | L | 789576 | Sweden, Stocholm Archipelago | 59°17' N, 18°28' E | 13.07.1950 | A. Gorter | A. Gorter/  AW |
| 35 |  | *suecica* | L | 789575-4 | DK, Mossø lake | 56°02' N, 09°46' E | 31.08.1960 | R. H. | R. H./AW |
| 36 |  | *suecica* L. | TRN | 620 | PL, Kołobrzeg | 54°07' N, 15°26' E | 28.08.1893 | P. Ascherson,  P. Graebner | -/AW |
| 37 |  | *suecica* L. | L | 932322 | GB, Scotland, Glenmore | 57°10' N, 03°41' W | 07.1976 | Ploeg | -/AW |
| 38 |  | *suecica* L. | KRAM | 2903 | SE, Torneträsk lake, Kopparåsen | 68°25' N, 18°36' E | 18.08.1945 | C. G. Alm | - |
| 39 |  | *suecica* L. | POZ | Brak danych | FI, Kuusamo | 65°57’ N, 29°11’ E | 15.07.1973 | K. Tobolski | -/AW |
| 40 |  | *suecica* L. | KOR | 46358 | FI, Laponia, Taivaskero mountain | 68°04’ N, 24°03’ E | 26.08.2008 | K. Boratyńska,  A. Boratyński | -/AW |
| 41 |  | - | BG1 | - | RU, Sachalin, Tihaja bay | 48°00’ N, 142°32’ E | No data | - | - |

* - *Index Herbariorum*, <http://sciweb.nybg.org/science2/IndexHerbariorum.asp>

Abbreviations: (BG1) - The Adam Mickiewicz University Botanical Garden; (BA) - Bosnia and Herzegovina; (CA) - Canada; (CN) – China; (CS) – Serbia and Montenegro; (FI)- Finland; (GB) - The British Isles; (DK) - Denmark; (GR) - Greece; (JP) – Japan; (KR) – South Korea;( PL) – Poland; (RU) – Russia; (SE) – Sweden; (SI) – Slovenia; (SK) - Slovakia; (US) - United States
