# Supplementary material for: The morphology and internal structure of dogwood (Cornus L.) endocarps in the taxonomy and phylogeny of the genus
Source: PeerJ. 2021 Oct 28;9:e12170. doi: 10.7717/peerj.12170 (PMC8557701; doi:10.7717/peerj.12170)
Supplement: Supplemental Information 4 [file peerj-09-12170-s004.docx]

Number of tested *Cornus* endocarps per specimens, species, subgenera, and groups

| *Cornus* taxa | Specimen | | | | | | | | | | | | | | | | | | | | | Number of endocarps |
| --- | --- | --- | --- | --- | --- | --- | --- | --- | --- | --- | --- | --- | --- | --- | --- | --- | --- | --- | --- | --- | --- | --- |
|  | 1 | 2 | 3 | 4 | 5 | 6 | 7 | 8 | 9 | 10 | 11 | 12 | 13 | 14 | 15 | 16 | 17 | 18 | 19 | 20 | 21 |  |
| *C. alba* | 5 | 5 | 5 | 5 | 6 | 17 | 30 | 18 | 22 | 12 | 30 | 30 | 30 | 30 | 30 |  |  |  |  |  |  | 275 |
| *C. alternifolia* | 5 | 5 | 2 | 3 | 5 | 10 | 30 | 30 | 30 | 30 | 30 | 10 |  |  |  |  |  |  |  |  |  | 190 |
| *C. amomum* | 5 | 5 | 6 | 10 | 30 | 30 | 30 | 30 | 30 | 30 |  |  |  |  |  |  |  |  |  |  |  | 206 |
| *C. australis* | 3 | 5 | 5 | 5 | 5 | 5 | 5 | 5 | 5 | 6 | 6 | 5 | 5 | 4 | 30 | 30 | 30 | 30 | 30 | 20 |  | 239 |
| *C. bretschneideri* | 30 | 30 | 30 |  |  |  |  |  |  |  |  |  |  |  |  |  |  |  |  |  |  | 90 |
| *C. controversa* | 5 | 5 | 11 | 3 | 5 | 5 | 3 | 3 | 30 | 30 | 30 | 30 | 30 | 30 |  |  |  |  |  |  |  | 220 |
| *C. drummondii* | 5 | 4 | 5 | 6 | 5 | 5 | 30 | 30 | 30 | 30 | 30 | 30 |  |  |  |  |  |  |  |  |  | 210 |
| *C. foemina* | 5 | 5 | 5 | 5 | 6 | 6 | 6 | 30 | 30 | 30 |  |  |  |  |  |  |  |  |  |  |  | 128 |
| *C. macrophylla* | 5 | 10 | 5 | 30 | 30 | 30 |  |  |  |  |  |  |  |  |  |  |  |  |  |  |  | 110 |
| *C. obliqua* | 6 | 5 | 5 | 7 | 8 | 30 | 30 | 30 | 30 | 30 |  |  |  |  |  |  |  |  |  |  |  | 181 |
| *C. occidentalis* | 5 | 3 | 3 | 5 | 5 | 4 | 3 | 6 | 30 | 30 | 30 |  |  |  |  |  |  |  |  |  |  | 124 |
| *C. racemosa* | 5 | 5 | 6 | 5 | 7 | 5 | 30 | 30 | 30 | 60 | 30 |  |  |  |  |  |  |  |  |  |  | 213 |
| *C. sanguinea* | 5 | 5 | 5 | 5 | 5 | 6 | 5 | 5 | 5 | 5 | 5 | 4 | 4 | 8 | 5 | 30 | 30 | 30 | 30 | 30 | 30 | 257 |
| *C. sericea* | 7 | 5 | 3 | 5 | 5 | 5 | 5 | 3 | 5 | 5 | 30 | 30 | 30 | 30 | 30 | 30 |  |  |  |  |  | 228 |
| *C. walteri* | 5 | 5 | 5 | 6 | 6 | 2 | 3 | 4 | 2 | 30 | 30 | 30 | 13 |  |  |  |  |  |  |  |  | 141 |
| *C. mas* | 3 | 5 | 5 | 4 | 3 | 30 | 30 | 30 | 30 | 30 | 30 |  |  |  |  |  |  |  |  |  |  | 200 |
| *C. officinalis* | 5 | 5 | 4 | 5 | 30 | 30 | 30 | 30 | 30 | 30 |  |  |  |  |  |  |  |  |  |  |  | 199 |
| *C. florida* | 4 | 5 | 5 | 6 | 30 | 30 | 30 | 30 | 21 | 20 |  |  |  |  |  |  |  |  |  |  |  | 181 |
| *C. kousa* | 3 | 7 | 8 | 8 | 30 | 30 | 30 | 30 | 25 | 19 |  |  |  |  |  |  |  |  |  |  |  | 190 |
| *C. nuttalii* | 5 | 3 | 6 | 5 | 2 | 24 |  |  |  |  |  |  |  |  |  |  |  |  |  |  |  | 45 |
| *C. canadensis* | 5 | 5 | 6 | 3 | 4 | 5 | 6 | 7 | 8 | 30 | 16 | 30 |  |  |  |  |  |  |  |  |  | 125 |
| *C. suecica* | 17 | 6 | 5 | 5 | 2 | 5 | 10 | 9 | 5 | 30 |  |  |  |  |  |  |  |  |  |  |  | 94 |
| **Total** |  |  |  |  |  |  |  |  |  |  |  |  |  |  |  |  |  |  |  |  |  | **3846** |
| *Kraniopsis* | 91 | 92 | 88 | 94 | 88 | 115 | 147 | 161 | 189 | 238 | 221 | 159 | 112 | 102 | 125 | 120 | 60 | 60 | 60 | 50 | 30 | 2402 |
| *Mesomora* | 10 | 10 | 13 | 6 | 10 | 15 | 3 | 3 | 30 | 30 | 60 | 60 | 60 | 60 | 30 | 10 |  |  |  |  |  | 410 |
| *Cornus* | 8 | 10 | 9 | 9 | 33 | 60 | 60 | 60 | 60 | 60 | 30 |  |  |  |  |  |  |  |  |  |  | 399 |
| *Cynoxylon* | 9 | 8 | 11 | 11 | 32 | 54 | 30 | 30 | 21 | 20 |  |  |  |  |  |  |  |  |  |  |  | 226 |
| *Syncarpea* | 3 | 7 | 8 | 8 | 30 | 30 | 30 | 30 | 25 | 19 |  |  |  |  |  |  |  |  |  |  |  | 190 |
| *Arctocrania* | 22 | 11 | 11 | 8 | 6 | 10 | 16 | 16 | 13 | 60 | 16 | 30 |  |  |  |  |  |  |  |  |  | 219 |
| **Total** |  |  |  |  |  |  |  |  |  |  |  |  |  |  |  |  |  |  |  |  |  | **3846** |
| BW | 101 | 102 | 101 | 100 | 98 | 130 | 150 | 164 | 219 | 268 | 281 | 219 | 172 | 162 | 155 | 130 | 60 | 60 | 60 | 50 | 30 | 2812 |
| CC | 8 | 10 | 9 | 9 | 33 | 60 | 60 | 60 | 60 | 60 | 30 |  |  |  |  |  |  |  |  |  |  | 399 |
| BB | 12 | 15 | 19 | 19 | 62 | 84 | 60 | 60 | 46 | 39 |  |  |  |  |  |  |  |  |  |  |  | 416 |
| DW | 22 | 11 | 11 | 8 | 6 | 10 | 16 | 16 | 13 | 60 | 16 | 30 |  |  |  |  |  |  |  |  |  | 219 |
| **Total** |  |  |  |  |  |  |  |  |  |  |  |  |  |  |  |  |  |  |  |  |  | **3846** |
